# Breath analysis based early gastric cancer classification from deep stacked sparse autoencoder neural network

**DOI:** 10.1038/s41598-021-83184-2

**Published:** 2021-02-17

**Authors:** Muhammad Aqeel Aslam, Cuili Xue, Yunsheng Chen, Amin Zhang, Manhua Liu, Kan Wang, Daxiang Cui

**Affiliations:** 1 Institute of Nano Biomedicine and Engineering, Shanghai Engineering Research Center for Intelligent Instrument for Diagnosis and Therapy, Department of Instrument Science & Engineering, School of Electronic Information and Electrical Engineering, Yantai Information Technology Research Institute of Shanghai Jiao Tong University, Shanghai Jiao Tong University, 800 Dongchuan Road, Shanghai, 200240 People’s Republic of China; 2 Department of Plastic and Reconstructive Surgery, Shanghai Ninth People’s Hospital, School of Medicine Shanghai Jiao Tong University, 639 Zhizaoju Road, Shanghai, 200011 People’s Republic of China; 3 National Engineering Research Center for Nanotechnology, 28 Jiangchuan Road, Shanghai, 200241 People’s Republic of China

**Keywords:** Computational biology and bioinformatics, Biomarkers, Health care, Medical research, Engineering

## Abstract

Deep learning is an emerging tool, which is regularly used for disease diagnosis in the medical field. A new research direction has been developed for the detection of early-stage gastric cancer. The computer-aided diagnosis (CAD) systems reduce the mortality rate due to their effectiveness. In this study, we proposed a new method for feature extraction using a stacked sparse autoencoder to extract the discriminative features from the unlabeled data of breath samples. A Softmax classifier was then integrated to the proposed method of feature extraction, to classify gastric cancer from the breath samples. Precisely, we identified fifty peaks in each spectrum to distinguish the EGC, AGC, and healthy persons. This CAD system reduces the distance between the input and output by learning the features and preserve the structure of the input data set of breath samples. The features were extracted from the unlabeled data of the breath samples. After the completion of unsupervised training, autoencoders with Softmax classifier were cascaded to develop a deep stacked sparse autoencoder neural network. In last, fine-tuning of the developed neural network was carried out with labeled training data to make the model more reliable and repeatable. The proposed deep stacked sparse autoencoder neural network architecture exhibits excellent results, with an overall accuracy of 98.7% for advanced gastric cancer classification and 97.3% for early gastric cancer detection using breath analysis. Moreover, the developed model produces an excellent result for recall, precision, and f score value, making it suitable for clinical application.

## Introduction

Gastric Cancer (GC) is one of the major types of cancer, deadliest, fourth commonest, and the second leading cancer-associated deaths worldwide^1–3^. According to the World Cancer Research Fund, China is among the top five countries, which have the highest rate of GC in 2018^4^. GC is associated with several factors such as lifestyle, genetic, and environment^5^. GC is classified into two stages: (1) AGC: Advanced Gastric Cancer, (2) EGC: Early Gastric Cancer. In clinics, endoscopy and biopsy (pathological examinations) are the most commonly used methods for the detection of AGC and EGC^6^. GC is a very aggressive type of malignancy, which is very difficult to detect at the early stages, due to vague symptoms of EGC^7,8^. Only in 2018, 1.8 million deaths were recorded due to poor prognosis of GC around the globe^9^. As the symptoms of GC at the early stages are not characteristic, most GC patients wasted precious time of treatment^10^. The mortality of gastric cancer can be reduced if it is diagnosed at early stages. Early prognosis is not only enough, but the mortality can be reduced by the prospective precise staging^11^. The mortality depends upon the early prognosis of the disease. AGC patients have 24% five-year survival rate, whereas EGC patients have 90% five-year survival rate^12^. Breathomics is an alternative noninvasive method, which can be used in the medical field to diagnose different diseases. Sometimes it is very difficult to reach a conclusion when a patient’s symptoms are complex and contradictory. The physician evaluates the observation and makes a decision depending on his understanding and analyzing the patient’s data.

In Greek, ancient physicians found odor in the breath to diagnose different diseases^13^. From the previous studies, it has been confirmed that breath gas is a complex mixture, which contains more than 3000 VOC biomarkers^14–16^. These VOCs change their properties during the metabolism, and hence can be used as cancer VOC biomarkers for the detection of GC^17^. Lung cancer and GC have been diagnosed by breath analysis^18^. In previous studies, authors have focused on the prewarning of different cancers.

Breath analysis is gaining attention in the diagnosis of the diseases, it is noninvasive in nature. Breath analysis can produce accurate and reproducible results without producing any harm to the patient during the diagnostic tests. VOCs are measured from the breath to distinguish patients from healthy populations. VOC biomarkers reflect cellular metabolite levels due to the disease states, which are transferred to the blood, urine, and saliva. These VOCs are responsible for the disease state discerned in the breath.

Computer Aided Diagnosis based techniques have been developed by computer scientists to help the physician in the course of making decision^19^. In general, the pathologists analyze the whole image to observe the abnormality in the specific cell/tissue. Moreover, the human eye is less adept to recognize these changes. Therefore, it is the need of time to develop sophisticated methods, which can help the pathologists to diagnose the disease with some ease. In this study, the authors have proposed a computer-aided based method using deep learning for breath analysis in gastric cancer classification, which can overcome the above-stated problems.

Deep learning is a subset of Artificial Intelligence^20^. Deep learning methods have been used for medical diagnosis, robotics, computer vision, bioinformatics, audio and speech recognition, industrial applications^21^. SSAE, CNN, DBN, and recurrent neural networks are some basic deep learning techniques, which have been used to achieve the state of art results for several tasks^22^. The success of many deep learning applications depends upon the big data, which is a prerequisite and converges the fields of data analytics and deep learning^23^. The use of Deep learning based algorithms has improved the accuracy of cancer prediction outcome 15–20%^24^.

The deep convolutional neural network is commonly used for two-dimensional data. Most of the deep learning based systems have been developed for breast cancer, very few authors have established systems for the prognosis of GC. Most of the authors have worked on medical images, which include histopathological images, PET images, X-Ray, MRI, and CT images^25^. A GC multistage detection system was developed by Oikawa et al., by using pathological images, which achieved a 14.1% false rate^26^. At the first stage, they used SVM to extract handicraft features at low resolution. In the next stage, CNN was developed for making the final decision. Another CNN based GC classification system for histopathological images was developed by Xu et al^27^. Their system was based on segmentation and classification. Firstly, they created small patches, and in the next stage, they used CNN to classify the epithelial and stromal tissues. Wang et al^28^ also presented a GC classification system, their work is very similar to Xu’s work. They also created patches, and in last they used CNN to classify these patches. Li et al^29^ proposed a system for the classification of GC using deep learning. They developed shallow and deep layers for the classification of GC. They achieved 100% accuracy for sliced based classification. They did not mention that how many of the patients belong to the early stage, as early-stage diagnosis is very important. Daniel et al^30^ proposed a GC classification system using VOC biomarker. They achieved an accuracy of 93%. They developed an Artificial Neural Network (ANN) using backpropagation algorithm. They also haven’t defined the EGC and AGC groups. Although, they achieved very good accuracy, still there is some room for better results. Few studies have already been carried out using miRNA, but their use in clinical applications is very limited, because of the low sensitivity^31–34^. Therefore, they cannot discriminate between benign and malignant samples at the early stages. Few authors have worked on gene expression signatures for the prognosis and detection of cancner^35–37^. These studies have potential, but at the same time, they have some limitations of microarray, due to which they cannot be preferred in the clinics.

Deep learning has already shown its effectiveness in several fields in recent years. The first deep autoencoder-based neural network was presented by Hinton et al^38^. It has resolved many different and challenging tasks related to histopathological image classification. Cruz et al. have proposed an architecture for the classification of cancer and non-cancerous regions. However, their autoencoder contains only one layer for the feature representation^39^. Xu et al. have developed a Stacked Sparse Autoencoder for Nuclei detection of Breast Cancer Histopathological images^40^. They used two-layer autoencoder for feature learning. Softmax classifier was used to classify the images. Feng et al., proposed a method, they extracted the features from the histopathological images by using deep manifold preserving autoencoder^41^. These features were learned from unlabeled data. Like Xu’s work, they used Softmax classifier in the last layer to classify the images. Their work is also based on breast cancer classification.

In this study, we have proposed and developed a deep learning based neural network that can distinguish between healthy people and cancerous patients. The proposed method can also distinguish between AGC and EGC. The diagnosis of EGC is very difficult, so we developed a CAD system to overcome this problem. The proposed deep stacked sparse autoencoder neural network architecture exhibits excellent results, with an overall accuracy of 96.3% for gastric cancer classification and 97.4% for early gastric cancer detection using breath analysis. The algorithm can be trained with less amount of time. Moreover, breath analysis based developed neural network has outperformed all the other existing techniques up to date.

### Results

In this work, we have developed several deep neural networks based on stacked sparse autoencoders. This study aims to develop a deep neural network architecture that can be used to distinguish early-stage gastric cancer patients from healthy persons. We developed and studied different schemes of hidden layers in each deep neural network to visualize and analyze the effect of it on feature extraction. Based on the investigation of different hidden layers schemes, we have evaluated the performance of each deep neural network.

We developed K-NN, Support Vector Machine, Linear Discriminant, and Decision Tree based neural networks for classification. The comparison of these developed neural networks and deep-stacked sparse neural network, we developed and investigated all the networks listed in Table 1. The proposed and developed deep stacked sparse autoencoder has outperformed all the other developed models of neural network. All the deep neural networks in this study consist of two hidden layers, each hidden layer comprises of different number of neurons. The objective of this study is to build a neural network architecture, which can classify the early-stage gastric cancer at high accuracy. The train + validate data was fixed at 70% and 15% respectively. Whereas, the test data was fixed to 15% of the total data.

**Table 1:** Comparison and performance evaluation of the different proposed deep stacked sparse autoencoder neural network.

Model number	Training accuracy	Test accuracy	Precision	F Score	Recall	AUC
SVM	–	–	67.7%	77.5%	90.8%	–
Decision Tree	–	–	58.7%	63.8%	70%	–
K-NN	–	–	70.9%	48.3%	36.7%	–
100 20	86.0%	69.1%	84.0%	85.9%	87.9%	0.9145
100 40	98.1%	89.1%	99.4%	99.4%	99.4%	0.9910
100 60	85.7%	83.6%	92.3%	88.3%	84.7%	0.9230
100 80	92.2%	76.4%	92.9%	94.76%	96.7%	0.9764
100 100	90.3%	87.3%	98.1%	96.8%	95.6%	0.9777
Chen et al.^1^	–	–	94.1%	89.9%	91.95^	–

The first model was developed with [100 20] size of autoencoders, the first hidden layer has 100 neurons and the second hidden layer carries 20 number of neurons. This model produces an overall accuracy of 81.5%. The second model was developed with [100 40] size of autoencoder, 100 and 40 are the number of neurons in the 1st and 2nd hidden layers respectively. This model produces an overall accuracy of 96.5%, this model misclassified only three samples of early-stage gastric cancer. Moreover, this model produces very good accuracy for predicting healthy people and advanced gastric cancer as well. This model provides an excellent result. This model yields an accuracy of 92.2%, 97.3% and 98.7% for EGC, Healthy, and AGC patients respectively. The third model was developed with [100 60] size of autoencoders, this model produces an overall accuracy of 84.2%. This model was unable to distinguish between gastric cancer patients and healthy persons more precisely. This model produces a misclassification of 12.7% in the healthy person class. The error rate was high in this class due to which this model can’t be used in clinical applications. The fourth and fifth models were developed with [100 80], [100 100] size of hidden layers respectively. These models produce an overall accuracy of 88.3% and 90.2% respectively.

Area Under the Curve (AUC) have been calculated to find the measure of performance across all possible classification thresholds. We have found the maximum AUC value for the second developed model, and the minimum value for AUC was obtained for the first developed model. All the AUC values for different models have been shown in Table 1.

The results from the Table 1 are indicating that the best classifier accuracy was 89.5% for the deep-stacked sparse autoencoder neural network (DSSAENN) with [100 40] size of the autoencoder. This model produces high results in Recall, Precision, F Score, and Detection rate as well. This model has outperformed all the other models developed during this study, and this model has produced more efficient results as compared to the previous studies. Therefore, this model can be used in clinical applications to help out clinical doctors reduce the mortality rate of gastric cancer. The minimum accuracy was achieved for [100 20] size of autoencoder based DSSAENN. The model developed with [100 100] size of autoencoder produces accuracy less than [100 40] based DSSAENN, but greater than all the other deep neural networks. Whereas, the models developed with [100 60] and [100 80] produces result better than the model [100 20] but locating behind the [100 40] and [100 100] based DSSAENN.

**Figure 1:**
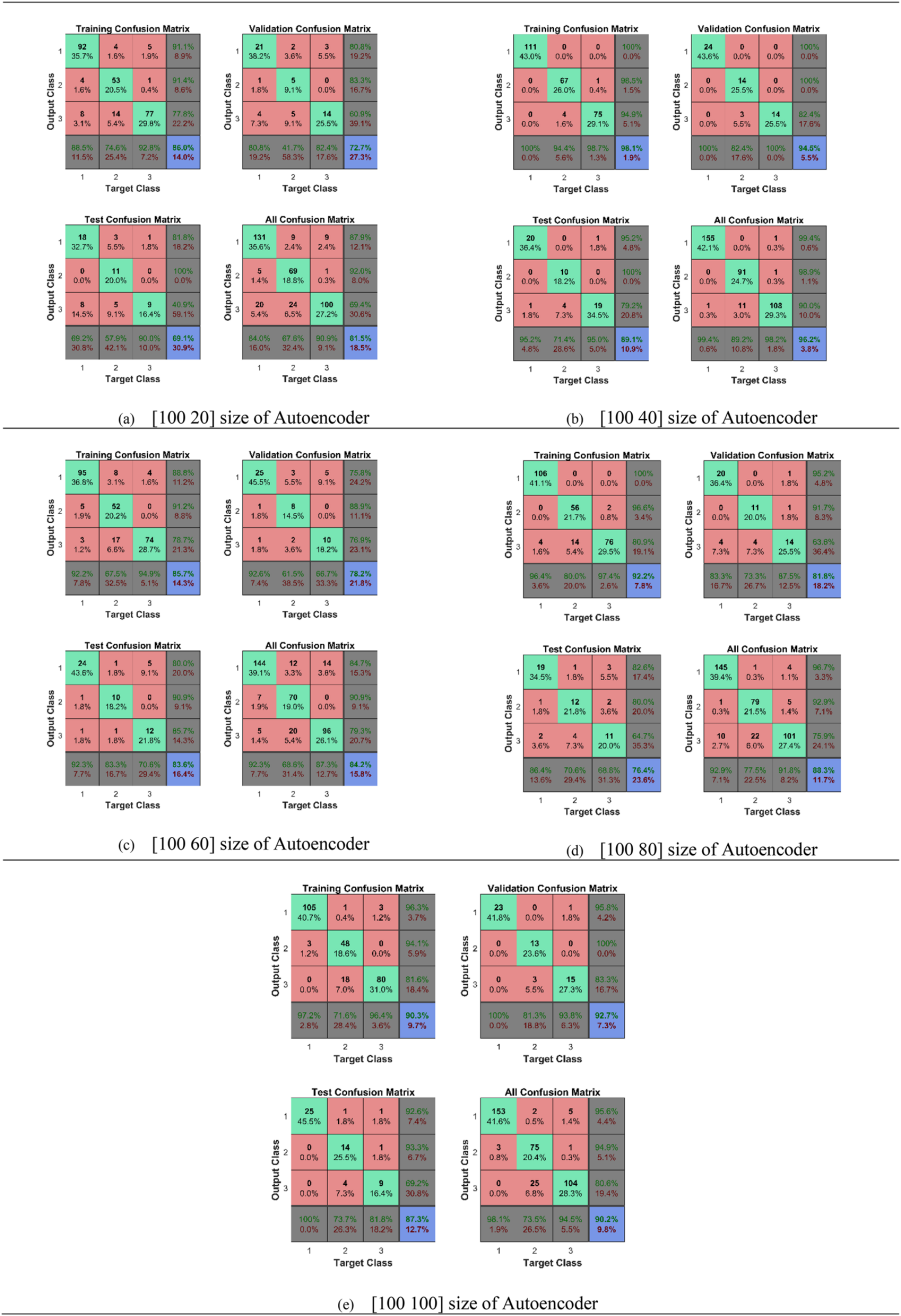
Confusion matrices of proposed deep stacked sparse autoencoder neural network with different number of neurons.

Figure 1 is representing the results in the form of confusion matrices obtained from the experiments. We trained the breath samples to distinguish the early-stage gastric cancer patients from the healthy persons with different neural networks. Figure 1, is indicating the training results of the different developed neural networks. In Fig. 1, the confusion matrices of training data, validation data, and test data have been shown separately. Whereas, the last confusion matrix of each in Fig. 1 is showing the overall accuracy result of that particular deep neural network.

**Figure 2:**
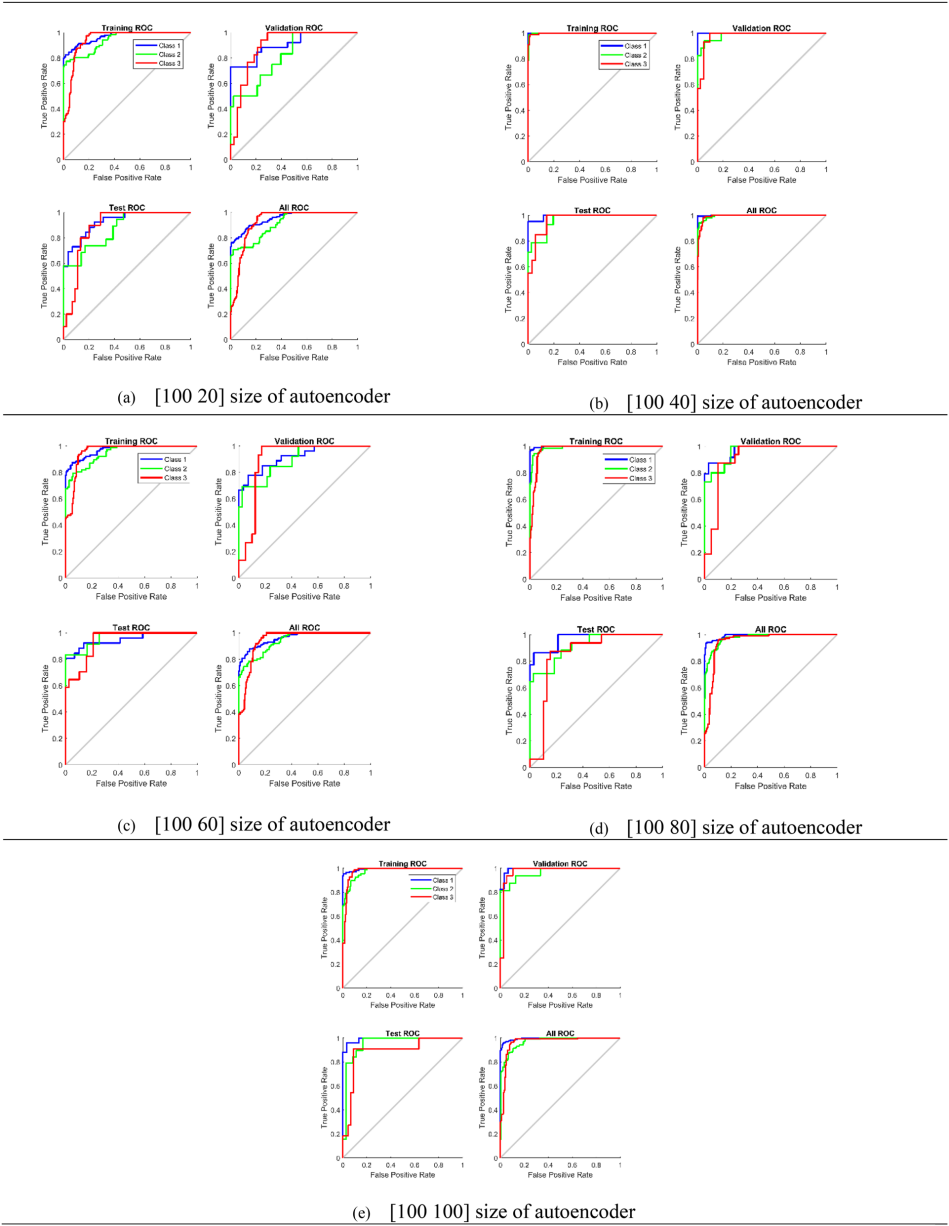
Receiver operating characteristics curves (ROCs) of proposed deep stacked sparse autoencoder neural network with different number of neurons.

Receiver Operating Characteristics (ROC) curve is an important tool for the evaluation of the neural network. The ROC curve for the DSSAENN is shown in Fig. 2. The ROC curves were used to visualize the performance of each developed deep neural network. These curves tell us about the compatibility of each model to distinguish between each class. The performance of the model is high if the area under the curve is more, and if the area under the curve is less this indicates poor performance of the model.

Figure 2a is representing the ROC curve obtained for the first deep neural network. This figure comprises four different ROCs. Which are the training ROC curve, Validation ROC curve, Test ROC curve, and All ROC curve respectively. Similarly, Fig. 2b–e are representing ROC curves for four other deep neural networks, which were [100 60], [100 40], [100 80] and [100 100] respectively. From Fig. 2, it can be observed that Fig. 2b has more area under the curve which means that this model has high performance. Whereas, Fig. 2a has the least area under the curve, giving a clear indication that this model has the lowest accuracy among the entire set of deep neural networks.

### Discussion

We have presented deep-stacked sparse autoencoder neural network architecture based model for the classification of EGC, AGC, and healthy persons from breath analysis. This model is capable of detecting gastric cancer at early stages with high accuracy and repeatability. The high-level features were captured in an unsupervised manner with the help of deep-stacked sparse autoencoder neural network. These high-level features make sure that the classifier can detect gastric cancer from the breath samples very effectively. The effectiveness of the deep-stacked sparse autoencoder neural network has been shown in Table 1. These high-level features were then incorporated with Softmax classifier (SMC) in a supervised manner to get the higher classification accuracy. The developed DSSAENN model for EGC detection is non-invasive, cheap, and faster as compared to the traditional gastric cancer detection methods. This method has provided an entirely new diagnostic way of EGC. This proposed and developed model can play an important part in clinical applications. The target and challenge of this study were to build a classifier based CAD system that can be used to detect gastric cancer patients at early stages by using breath samples. The proposed model is precise and reliable. The overall performance of the developed model is very high. From the experiments, our developed neural network architecture, DSSAENN outperforms the previous studies. The developed model produces an overall accuracy of 99.2% for training data, 89.5% for validation data, 89.5% for test data. This model produces an overall accuracy for the detection of EGC to 97.4%, AGC 93.3%, and healthy person 98%making an overall accuracy of the developed model owns clinical translation prospect.

In this study, we have used autoencoder to extract the features from breath. In near future, we will extract the features by using Convolutional Neural Network (CNN). Feature extraction plays an important role in the performance of the neural network. Subsequently, a Computer-Aided Design system based on Deep Stacked Sparse Autoencoder Neural Network using Field Programmable Gate Array or other embedded systems are still an exciting task, and the hardware application of such systems can support medical professionals in the diagnosis of EGC. We are also developing a communication link so that we can use the standalone application in remote areas as well^42^. In near future, we will develop a neural network on a single chip combined with breath diagnosis sensors to diagnose precisely the early gastric cancer in the remote areas via internet of things.

## Materials and methods

### Patients

This study was carried out under the guidelines of Reporting Recommendations for Tumor Marker Prognostic Studies (REMARK). All the breath samples were collected from the Shanghai Tongren Hospital, Shanghai, China. All the individuals were already guided about the conduction of clinical research. This study was approved by ethics committee of Shanghai Jiao Tong university. There were 200 volunteers, which include 55 EGC patients, 56 healthy persons, and 89 AGC patients. There were three criteria which were followed while collecting the breath samples, (1) individuals have already gone through the clinical diagnosis of GC using different techniques, either biopsy or endoscopy; (2) excluding the patients with other malignancies; (3) excluding the patients with metabolic diseases, mainly including diabetes. Table 2 shows the clinical characteristics of the volunteers.

**Table 2:** Clinical characteristics of volunteers.

Group	Number	Age (years)	Gender (M:F)	Tobacco consumption
Healthy	56	30 ± 7	35:21	25%
EGC	55	48.6 ± 12.1	40:15	36%
AGC	89	56.3 ± 11.7	68:21	45%

The AJCC Cancer Staging Manual was used for the GC stages. Age and gender have no impact on the EGC patients, AGC patients, and healthy person, therefore, we have excluded this information to make the resulting bias less. All the volunteers were asked to clean the mouth and refrain from eating and drinking for about 1 h. We assigned 75% breath samples for the training set and 25% breath samples were used for the validation set. In this study, we have used spectral region from 400 to 1500 nm for modeling.

### Data augmentation

The stability of any neural network depends on how well the model has learn the internal characteristics of the input data. As the number of samples increases, the stability of the neural network also increases. A sufficient amount of data is needed to avoid the overfitting and under fitting problems. In this study, we used data augmentation technique to produce additional data. The input to the proposed architecture is one dimensional, it contains the entire spectrum. This size is very large. Therefore, we crop the spectrum and used 1200 different values of each spectrum, 200 spectra are in total. Dataset can be expanded by moving breath samples either to right or left. In this study, we have shifted the breath samples on the right by 2 cm^−1^. There were 1200 spectral values for each breath sample and total number of spectral values were 240,000, after performing the data augmentation the number of samples were 368 and total number of spectral values for input data was 453,600.

### Data preprocessing

The breath samples were collected from the hospital, and these samples were affected by some noise. The data preprocessing steps were carried out on these breath samples to make them useful. This noise factor may lead us to the wrong classification. We eliminate and reduce the irrelevant and random variations from the breath samples.

Spikes were generated, as the breath sample hits the detector. These spikes have narrow bandwidth with positive peaks. These spikes are random in nature and produced due to different position on the sensors. The bandwidth of spike is very small as compare to the Raman spectra. We remove the spikes from the Raman samples by using the above assumption. The noise has high frequency. There are several techniques which have been proposed to remove noise from the data. In this study, we have used median filter to remove noise from breath samples. The medial filter eliminates the noise effectively.

Baseline correction is an essential part of preprocessing to avoid the left over background problem, which is produced due to the negative values of the spectra. The baseline correction does not cut down Raman band signal strength. Labspec5 software was used for the baseline correction of each spectrum, and smoothing the spectrum was also carried out by the Labspec5 software.

### Feature extraction

We defined a total of 1200 breath features from the breath samples of each individual. The Raman spectral feature includes Raman spectral pattern, band numbers, peak positions, peak width, area, and so on. These parameters play an important part in the interpretation of the Raman spectra. We extracted dominant peaks with in-house developed data analysis software incorporated in MATLAB 2017b. We identified fifty peaks in each sample. These fifty peaks were used as an input to the proposed Deep Stacked Sparse Autoencoder Neural Network (DSSAENN) to train the desired model.

### Autoencoder (AE)

**Figure 3:**
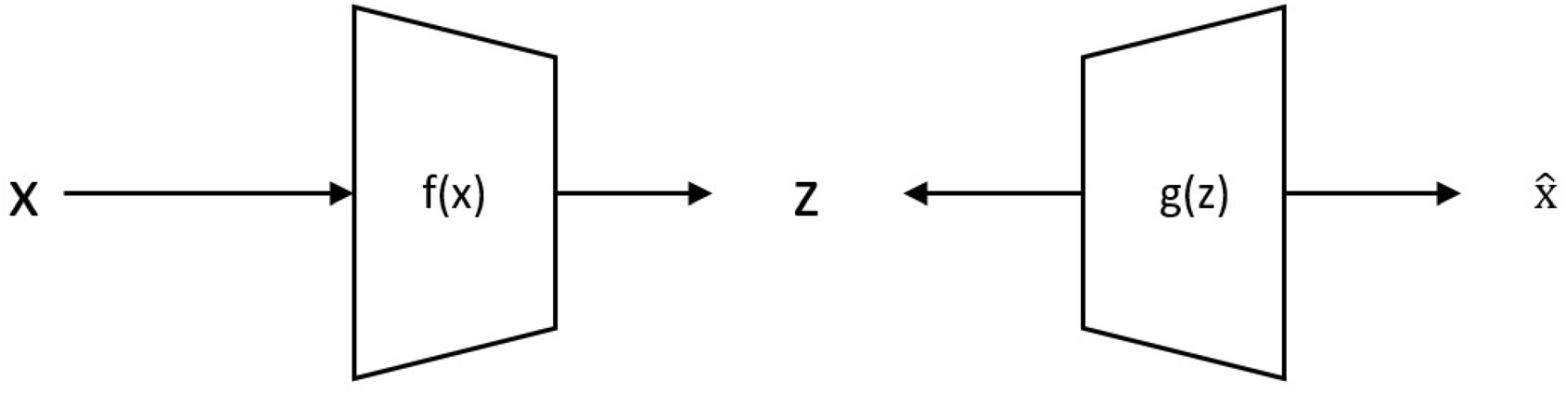
Basic structure of autoencoder.

Autoencoder is an unsupervised machine learning tool. It develops a better feature representation for the high dimensional input data. It finds the correlation between the input data. It is a multilayer feed-forward neural network, which represents the input with backpropagation. Figure 3 shows the basic structure of an autoencoder. Autoencoder minimizes the differences between the input and reconstructed data with the help of backpropagation.

1 $$f\left( x \right) = s\left( {wx + b} \right) = z$$

2 $$g\left( z \right) = s\left( {w^{\prime}z + b^{\prime}} \right) = \hat{x}$$

3 $$h\left( x \right) = g\left( {f\left( x \right)} \right) = \hat{x}$$

Here x is the input data, z is some latent representation, s is an activation function, f denotes the encoder function and g denotes the decoder function, w is the weight, h is an approximation of the identity function and b represents the biases values.

### Basic Sparse Autoencoder (SAE)

**Figure 4:**
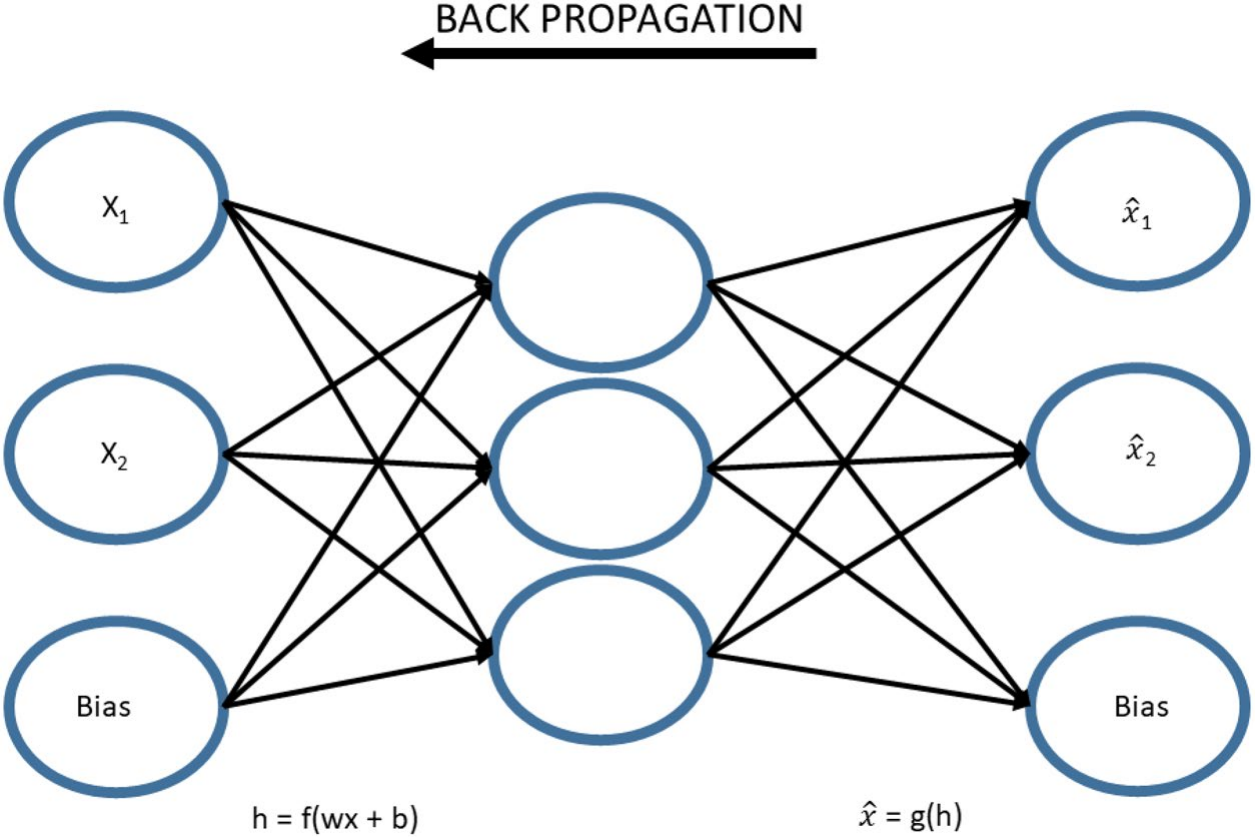
Basic architecture of sparse autoencoder.

The basic structure of Sparse Autoencoder (SAE) for high-level feature learning of breath analysis is shown in Fig. 4. SAE learned high dimensional structured features representation of cancerous or non-cancerous data by using an unsupervised feature learning algorithm. The input x was transformed into h by corresponding representation, at the input layer of the encoder. The hidden layer h visualizes the input data with new features. The hidden representation h was reconstructed from the new input data $\hat{x}$, this was done by the decoder at the output layer. The minimum discrepancy was found out by training the autoencoder between input x and its reconstructed value $\hat{x}$, to attain the optimal parameter values. The discrepancy was achieved with the use of backpropagation algorithm. The cost function of SAE is shown in Eq. (4), which comprises of three terms^42,43^.

4 $$J_{T} \left( {W,b} \right) = \frac{1}{n}\mathop \sum \limits_{i = 1}^{n} L\left( {x\left( i \right),y\left( i \right)} \right) + + \mathop \sum \limits_{j = 1}^{n} KL (p||\acute{\rho}_{j} ) + \beta \left\| W \right\|_{2}^{2}$$

The discrepancy between input and its reconstructed representation is shown by the first term, which is the average sum of square error. In the second term of Eq. (4), n represents the number of hidden layers and index j is summing the network over hidden units. The parameter ɑ shows the sparsity value. In general, this value is approximately zero but not equal to zero. The target activation of h is represented by $p$ and the average activation of j-th hidden unit over n training data is denoted by ῤ. This is calculated by the following formula.

5 $$\acute{\rho} = \frac{1}{n}\mathop \sum \limits_{i = 1}^{N} h_{j} \left( i \right)$$

KL (p||ῤ_j_) is the Kullback–Leibler divergence function^43^, which was define as KL(p||ῤ_j_) = plog p/ῤ_j_ + (1-p) log (1-p) / (1-ῤ_j_). The difference of two different distributions is measured by KL divergence function. The third term helps in overfitting the model, weight decay. This term tries to decrease the weight.

6 $$\parallel W\parallel_{2}^{2} = tr\left( {W^{T} W} \right) = \mathop \sum \limits_{l = 1}^{nl} 1\mathop \sum \limits_{i}^{nl - 1} 1\mathop \sum \limits_{j}^{sl} \left( {w_{i,j}^{l} } \right)^{2}$$

Here, the number of layers and number of neurons in first layer is represent by nl and sl respectively. The connection between j-th neuron of l-1 layer and j-th neuron of l layer is shown by the term ${\varvec{w}}_{{{\varvec{i}},{\varvec{j}}}}^{{\left( {\varvec{l}} \right)}}$.

Let X = {x(1),x(2),x(3),x(4),x(5),x(6),…,x(N)}^T^ is the entire unlabeled dataset, used for training in this study. Here x(k) ϵ $R^{{d_{n} }}$, N is the total number of breath samples and d_n_ is the total number of attributes in each breath sample. The learned high level features at 1st layer are represented as h^(1)^(k) = $\{ h_{1}^{1} \left( k \right),h_{2}^{1} \left( k \right),h_{3}^{1} \left( k \right),h_{4}^{1} \left( k \right), h_{5}^{1} \left( k \right),h_{6}^{1} \left( k \right), \ldots \ldots ..,h_{{d_{n} }}^{1} \left( k \right)\}^{T} ,$ for k-th breath sample, d_k_ represents the hidden units at the 1st layer. We used superscript notation to define hidden layers and subscript notation to define units in the whole manuscript. From the following figure, $h_{j}^{1} ,$ indicates the j-th unit in the 1st layer. For simplicity, x and $h^{1}$ denotes the input breath sample and its representation at 1st layer respectively.

### Stacked Sparse Autoencoder (SSAE)

**Figure 5:**
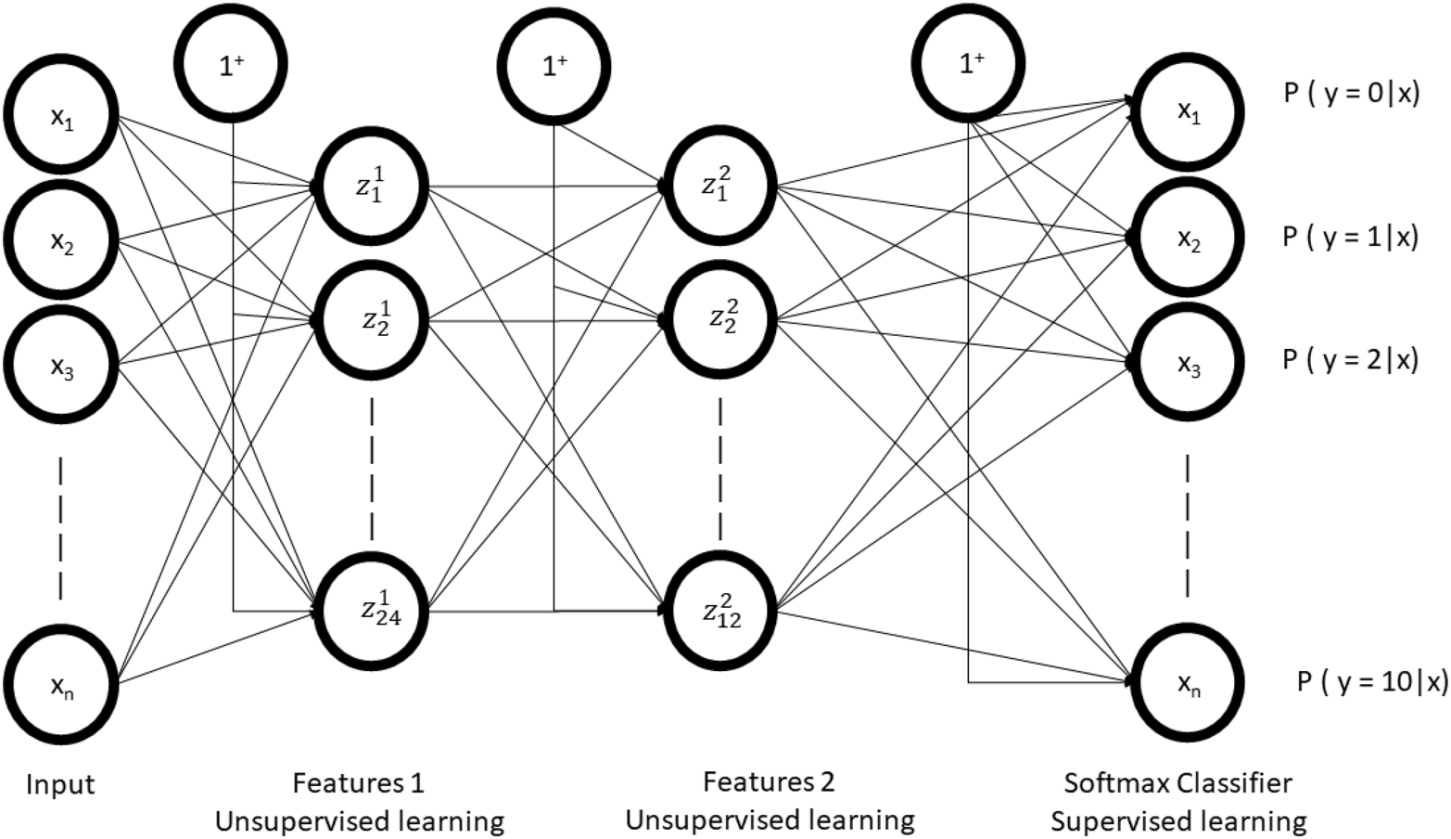
Block diagram of stacked sparse autoencoder with SMC classifier.

We developed a stacked sparse autoencoder by cascading multiple layers of basis sparse autoencoder. The output of each layer was fed as an input to the successive layer. In this study, we constructed two layers of sparse autoencoder to develop two layers stacked sparse autoencoder neural network. The basic structure of the stacked sparse autoencoder neural network has shown in Fig. 5. The first layer is the input layer, the last layer is called the output layer, the hidden layers work as a bridge between the input layer to the output layer. There were d_x_ = 50 * 200 input units in the input layer. The first hidden layer has d_h(1)_ = 100 units and the second layer has d_h(2)_ = 50 units as well.

### Softmax layer

Stacked autoencoders have trained each layer of the network using unlabeled data, as SAE belongs to the unsupervised learning algorithm category. The reconstruction of the input has provided by a feature vector. This feature vector will feed into the classifier so that the classification of the stacked sparse auto-encoder’s input data. Logistic regression is commonly used for supervised classification, where we have one or two classes at the output. Since we have three classes at the output, we cannot use logistic regression and we used Softmax classifier because of its multiclass classification property. Softmax classification is the modified form of the logistic regression whose function is to generalize the logistic regression.

7 $$f_{w} = 1/(1 + \exp \left( { - xw^{T} } \right)$$

here f(.) is the sigmoid function, and w is the input weights. Softmax layer is present, just before the output layer. This layer allows the output to be interpreted directly as probability.

### Deep stacked sparse autoencoder neural network

**Figure 6:**
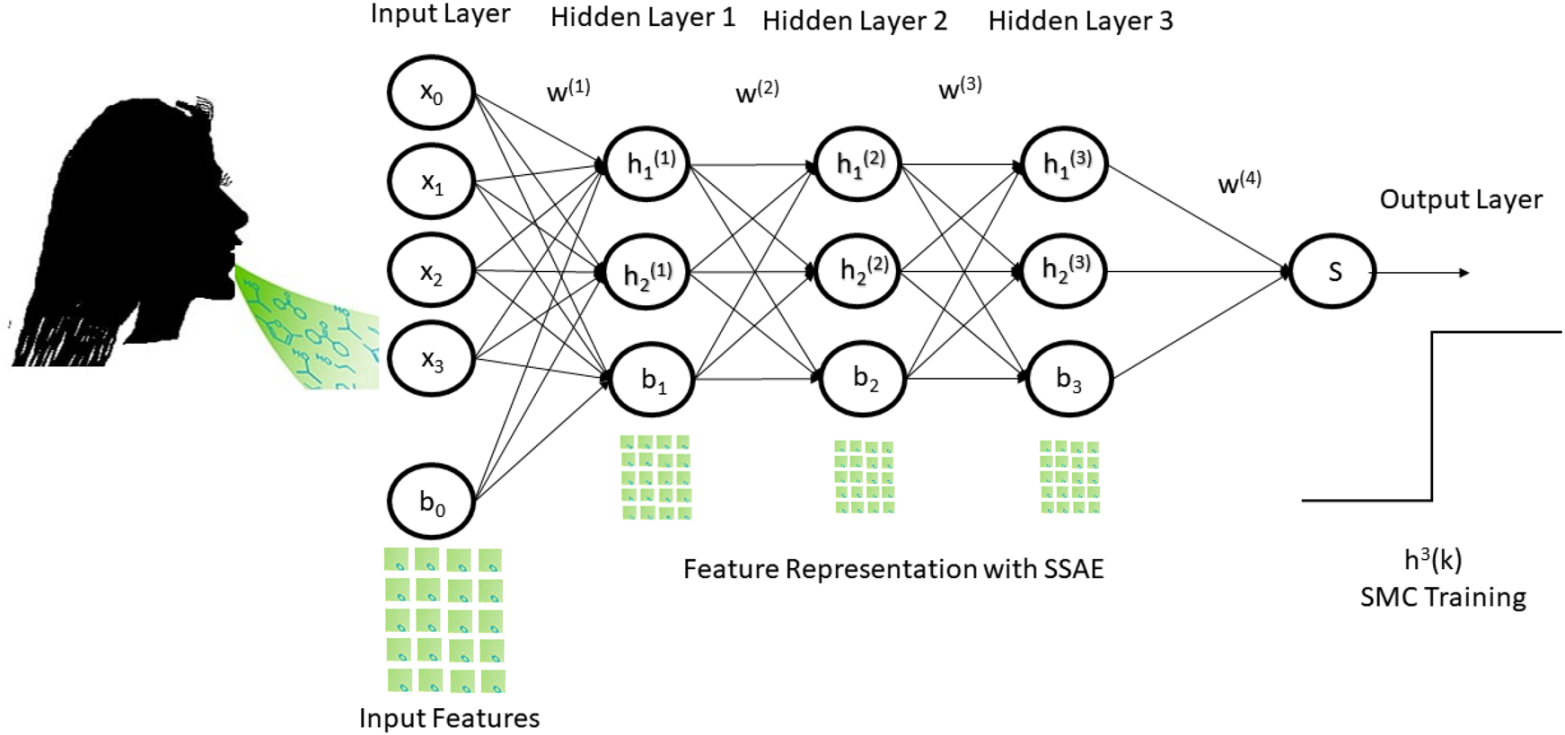
Proposed architecture of DSSAENN.

Figure 6 shows the complete architecture of this study. The complete architecture of this neural network comprises of two sparse autoencoder. The output of SSAE was wired into the Softmax layer.

### Implementation

The proposed deep neural network was developed and tested using MATLAB 2017b (Math Works, Natick, MA, United States) for the classification of gastric cancer. The network was trained on Core i5-2350 M CPU, 2.3 GHz. The initial learning rate was set to 0.0001 after trivial methods. The neural network was converged after 1000 epochs. The values of all the parameters for this study have been shown in Table 3.

**Table 3:** Parameters for proposed deep stacked sparse autoencoder neural network.

Model number	Learning rate		Hidden size		Sparsity regularization		Sparsity proportion	
	1st AE	2nd AE	1st AE	2nd AE	1st AE	2nd AE	1st AE	2nd AE
1	0.001	0.001	100	20	4	4	0.05	0.05
2	0.001	0.001	100	40	4	4	0.05	0.05
3	0.001	0.001	100	60	4	4	0.05	0.05
4	0.001	0.001	100	80	4	4	0.05	0.05
5	0.001	0.001	100	100	4	4	0.05	0.05

### Performance evaluation

This study aims to develop a classifier that can distinguish between the EGC, AGC, and healthy person. We developed DSSAENN and compared this method against the two other methods: (1) Softmax classifier (2) SAE + Softmax Classifier. Softmax classifier was used to classify the raw data. Whereas, in SAE + SMC based neural network, the features were learned by the SAE and these features act as raw input to the Softmax classifier, which was used to classify the EGC, AGC, and healthy persons. In this study, GC classification is a three-class problem. Three possible results can occur at the outcome of the classifier 0,1 and 2, which were regarded as EGC, AGC, and healthy respectively. The classification results of each developed model were calculated in terms of F1 score, Recall, specificity, sensitivity, and detection rate.

8 $$Precision = \frac{TP}{{TP + FP }}$$

9 $$Recall = \frac{TP}{{\left( {TP + FN} \right)}}$$

10 $$F{\mathrm{-}}Score = 2*\frac{Precision*Recall}{{Precision + Recall}}$$

11 $$Accuracy = \frac{TN + TP}{{TP + TN + FP + FN}}$$

Here TP, TN, FP and FN are known as true positive, true negative, false positive, and false negative respectively. For a good classifier, the model should have high accuracy, but at the same time, the precision and recall should also be minimum^44^. If any of the above criteria does not fulfill, the designed model is not accurate and it cannot be used in clinical applications.

### Informed consent

Informed consent was obtained from all individual participants included in this study.

## Data Availability

Dataset used in this particular study can be obtained from the corresponding author on reasonable request.

## References

[1] Chen Y, Zhang Y, Pan F, Liu J, Wang K, Zhang C, Cheng S, Lu L, Zhang W, Zhang Z, Zhi X (2016). Breath analysis based on surface-enhanced Raman scattering sensors distinguishes early and advanced gastric cancer patients from healthy persons. ACS Nano.

[2] Pourhoseingholi MA, Vahedi M, Baghestani AR (2015). Burden of gastrointestinal cancer in Asia; an overview. Gastroenterol. Hepatol. Bed Bench.

[3] Jing JJ, Liu HY, Hao JK, Wang LN, Wang YP, Sun LH, Yuan Y (2012). Gastric cancer incidence and mortality in Zhuanghe, China, between 2005 and 2010. World J. Gastroenterol.: WJG.

[4] Ferlay J, Colombet M, Soerjomataram I (2018). Global and Regional Estimates of the Incidence and Mortality for 38 Cancers: GLOBOCAN 2018.

[5] Ooki A, Yamashita K, Kikuchi S, Sakuramoto S, Katada N, Watanabe M (2009). Phosphatase of regenerating liver-3 as a prognostic biomarker in histologically node-negative gastric cancer. Oncol. Rep..

[6] Chen Y, Cheng S, Zhang A, Song J, Chang J, Wang K, Zhang Y, Li S, Liu H, Alfranca G, Aslam MA, Cui DX (2018). Salivary analysis based on surface enhanced Raman scattering sensors distinguishes early and advanced gastric cancer patients from healthy persons. J. Biomed. Nanotechnol..

[7] Axon A (2006). Symptoms and diagnosis of gastric cancer at early curable stage. Best Pract. Res. Clin. Gastroenterol..

[8] Sheikh IA, Mirza Z, Ali A, Aliev G, Md Ashraf G (2016). A proteomics based approach for the identification of gastric cancer related markers. Curr. Pharm. Des..

[9] Bray F, Ferlay J, Soerjomataram I, Siegel RL, Torre LA, Jemal A (2018). Global cancer statistics 2018: GLOBOCAN estimates of incidence and mortality worldwide for 36 cancers in 185 countries. CA Cancer J. Clin..

[10] Yazici O, Sendur MAN, Ozdemir N, Aksoy S (2016). Targeted therapies in gastric cancer and future perspectives. World J. Gastroenterol..

[11] Liao SR, Dai Y, Huo L, Yan K, Zhang L, Zhang H, Gao W, Chen MH (2004). Transabdominal ultrasonography in preoperative staging of gastric cancer. World J. Gastroenterol.: WJG.

[12] Alberts SR, Cervantes A, Van de Velde CJH (2003). Gastric cancer: epidemiology, pathology and treatment. Ann. Oncol..

[13] Kim KH, Jahan SA, Kabir E (2012). A review of breath analysis for diagnosis of human health. TrAC, Trends Anal. Chem..

[14] Hakim M, Broza YY, Barash O, Peled N, Phillips M, Amann A, Haick H (2012). Volatile organic compounds of lung cancer and possible biochemical pathways. Chem. Rev..

[15] Amal H, Ding L, Liu BB, Tisch U, Xu ZQ, Shi DY, Zhao Y, Chen J, Sun RX, Liu H, Ye SL (2012). The scent fingerprint of hepatocarcinoma: in-vitro metastasis prediction with volatile organic compounds (VOCs). Int. J. Nanomed..

[16] Peng G, Hakim M, Broza YY, Billan S, Abdah-Bortnyak R, Kuten A, Tisch U, Haick H (2010). Detection of lung, breast, colorectal, and prostate cancers from exhaled breath using a single array of nanosensors. Br. J. Cancer.

[17] Konvalina G, Haick H (2014). Sensors for breath testing: from nanomaterials to comprehensive disease detection. Acc. Chem. Res..

[18] Xu ZQ, Broza YY, Ionsecu R, Tisch U, Ding L, Liu H, Song Q, Pan YY, Xiong FX, Gu KS, Sun GP (2013). A nanomaterial-based breath test for distinguishing gastric cancer from benign gastric conditions. Br. J. Cancer.

[19] Güvenir HA, Emeksiz N, Ikizler N, Örmeci N (2004). Diagnosis of gastric carcinoma by classification on feature projections. Artif. Intell. Med..

[20] LeCun Y, Bengio Y, Hinton G (2015). Deep learning. Nature.

[21] Deng L, Yu D (2014). Deep learning: methods and applications. Found. Trends in Signal Process..

[22] Deng, L. Three classes of deep learning architectures and their applications: a tutorial survey. In *APSIPA Transactions on Signal and Information Processing* (2012).

[23] Sharma H, Zerbe N, Klempert I, Hellwich O, Hufnagl P (2017). Deep convolutional neural networks for automatic classification of gastric carcinoma using whole slide images in digital histopathology. Comput. Med. Imaging Graph..

[24] Cruz JA, Wishart DS (2006). Applications of machine learning in cancer prediction and prognosis. Cancer Inform..

[25] Kaucha, D.P., Prasad, P.W.C., Alsadoon, A., Elchouemi, A. & Sreedharan, S. Early detection of lung cancer using SVM classifier in biomedical image processing. In *2017 IEEE International Conference on Power, Control, Signals and Instrumentation Engineering (ICPCSI)*, 3143–3148, IEEE, September (2017).

[26] Oikawa K, Saito A, Kiyuna T, Graf HP, Cosatto E, Kuroda M (2017). Pathological diagnosis of gastric cancers with a novel computerized analysis system. J. Pathol. Inform..

[27] Xu J, Luo X, Wang G, Gilmore H, Madabhushi A (2016). A deep convolutional neural network for segmenting and classifying epithelial and stromal regions in histopathological images. Neurocomputing.

[28] Wang, D., Khosla, A., Gargeya, R., Irshad, H. & Beck, A.H. *Deep learning for identifying metastatic breast cancer*. arXiv preprint, http://arxiv.org/1606.05718 (2016).

[29] Li, Y., Li, X., Xie, X. & Shen, L. Deep learning based gastric cancer identification. In *2018 IEEE 15th International Symposium on Biomedical Imaging (ISBI 2018)*, 182–185, IEEE, April (2018).

[30] Daniel DAP, Thangavel K (2016). Breathomics for gastric cancer classification using back-propagation neural network. J. Med. Signals Sens..

[31] Fortunato O, Boeri M, Verri C, Conte D, Mensah M, Suatoni P, Pastorino U, Sozzi G (2014). Assessment of circulating microRNAs in plasma of lung cancer patients. Molecules.

[32] Heneghan HM, Miller N, Kerin MJ (2010). MiRNAs as biomarkers and therapeutic targets in cancer. Curr. Opin. Pharmacol..

[33] Madhavan, D., Cuk, K., Burwinkel, B. and Yang, R. Cancer diagnosis and prognosis decoded by blood-based circulating microRNA signatures. Frontiers in genetics, 4, (2013) 10.3389/fgene.2013.00116 PMC3689027 23802013

[34] Zen K, Zhang CY (2012). Circulating microRNAs: a novel class of biomarkers to diagnose and monitor human cancers. Med. Res. Rev..

[35] Koscielny S (2010). Why most gene expression signatures of tumors have not been useful in the clinic. Sci. Transl. Med..

[36] Michiels S, Koscielny S, Hill C (2005). Prediction of cancer outcome with microarrays: a multiple random validation strategy. The Lancet.

[37] Guyon I, Weston J, Barnhill S, Vapnik V (2002). Gene selection for cancer classification using support vector machines. Mach. Learn..

[38] Hinton GE, Salakhutdinov RR (2006). Reducing the dimensionality of data with neural networks. Science.

[39] Cruz-Roa, A.A., Ovalle, J.E.A., Madabhushi, A. & Osorio, F.A.G. A deep learning architecture for image representation, visual interpretability and automated basal-cell carcinoma cancer detection. In *International Conference on Medical Image Computing and Computer-Assisted Intervention*, 403–410, (Springer, Berlin, Heidelberg, 2013) 10.1007/978-3-642-40763-5_50 24579166

[40] Xu J, Xiang L, Liu Q, Gilmore H, Wu J, Tang J, Madabhushi A (2015). Stacked sparse autoencoder (SSAE) for nuclei detection on breast cancer histopathology images. IEEE Trans. Med. Imaging.

[41] Feng Y, Zhang L, Mo J (2018). Deep manifold preserving autoencoder for classifying breast cancer histopathological images. IEEE/ACM Trans. Comput. Biol. Bioinf..

[42] Ranzato MA, Poultney C, Chopra S, Cun Y (2006). Efficient learning of sparse representations with an energy-based model. Adv. Neural. Inf. Process. Syst..

[43] Kullback S, Leibler RA (1951). On information and sufficiency. Ann. Math. Stat..

[44] Jin H, Yu JK, Lin SJ (2020). Nanosensor-based flexible electronic assisted with light fidelity communicating technology for volatolomics-based telemedicine. ACS Nano.

